# Funding Infectious Disease Research: A Systematic Analysis of UK Research Investments by Funders 1997–2010

**DOI:** 10.1371/journal.pone.0105722

**Published:** 2014-08-27

**Authors:** Joseph R. Fitchett, Michael G. Head, Mary K. Cooke, Fatima B. Wurie, Rifat Atun

**Affiliations:** 1 King’s College London, Department of Infectious Diseases, London, United Kingdom; 2 University College London, Department of Infection and Population Health, UCL Royal Free Campus, London, United Kingdom; 3 Imperial College Business School and the Faculty of Medicine, Imperial College London, South Kensington Campus, London, United Kingdom; 4 Harvard School of Public Health, Harvard University, Boston, Massachusetts, United States of America; Brunel University, United Kingdom

## Abstract

**Background:**

Research investments are essential to address the burden of disease, however allocation of limited resources is poorly documented. We systematically reviewed the investments awarded by funding organisations to UK institutions and their global partners for infectious disease research.

**Methodology/Principal Findings:**

Public and philanthropic investments for the period 1997 to 2010 were included. We categorised studies by infectious disease, cross-cutting theme, and by research and development value chain, reflecting the type of science. We identified 6165 funded studies, with a total research investment of UK £2.6 billion. Public organisations provided £1.4 billion (54.0%) of investments compared with £1.1 billion (42.4%) by philanthropic organisations. Global health studies represented an investment of £928 million (35.7%). The Wellcome Trust was the leading investor with £688 million (26.5%), closely followed by the UK Medical Research Council (MRC) with £673 million (25.9%). Funding over time was volatile, ranging from ∼£40 million to ∼£160 million per year for philanthropic organisations and ∼£30 million to ∼£230 million for public funders.

**Conclusions/Significance:**

Infectious disease research funding requires global coordination and strategic long-term vision. Our analysis demonstrates the diversity and inconsistent patterns in investment, with volatility in annual funding amounts and limited investment for product development and clinical trials.

## Introduction

Since 2000, there has been substantial increase in international financing for global health from donor governments and innovative financing, in particular for infectious diseases.[1]

While the Organization for Economic Cooperation and Development (OECD) tracks donor contributions to overseas development assistance for health, including for selected infectious diseases, there are no internationally adopted systems for tracking innovative financing^1^ or investments in infectious disease research for addressing global health burden, by countries, or by funding entities. To date, few studies have analysed research and development (R&D) investments.[2]–[3]

Annual global research and development (R&D) funding for neglected diseases,[4] and funding by the National Institutes of Health (NIH) of the United States (US) Department of Health and Human Services have been estimated for selected years.[5]–[6] A recent systematic analysis of infectious disease research investments in the United Kingdom (UK) from 1997 to 2010 and burden of disease in 2004 and 2008 revealed mismatches between the amounts of funds invested and the burden of disease caused by the conditions, raising concerns about the efficiency of allocation of the investments in infectious disease R&D.^2^,^7^

The World Health Organization (WHO) Consultative Expert Working Group on Research and Development: Financing and Coordination is currently reviewing the feasibility of establishing a global observatory to monitor R&D investments.[8]–[11] The initiative was endorsed by member states at the sixty-sixth World Health Assembly this year.

We present the first systematic and comprehensive analysis of investments in infectious disease R&D over the 14-year period from 1997 to 2010. Specifically, the analysis focuses on investment patterns by global health institutions funding infectious disease research.

## Methods

**Figure 1:**
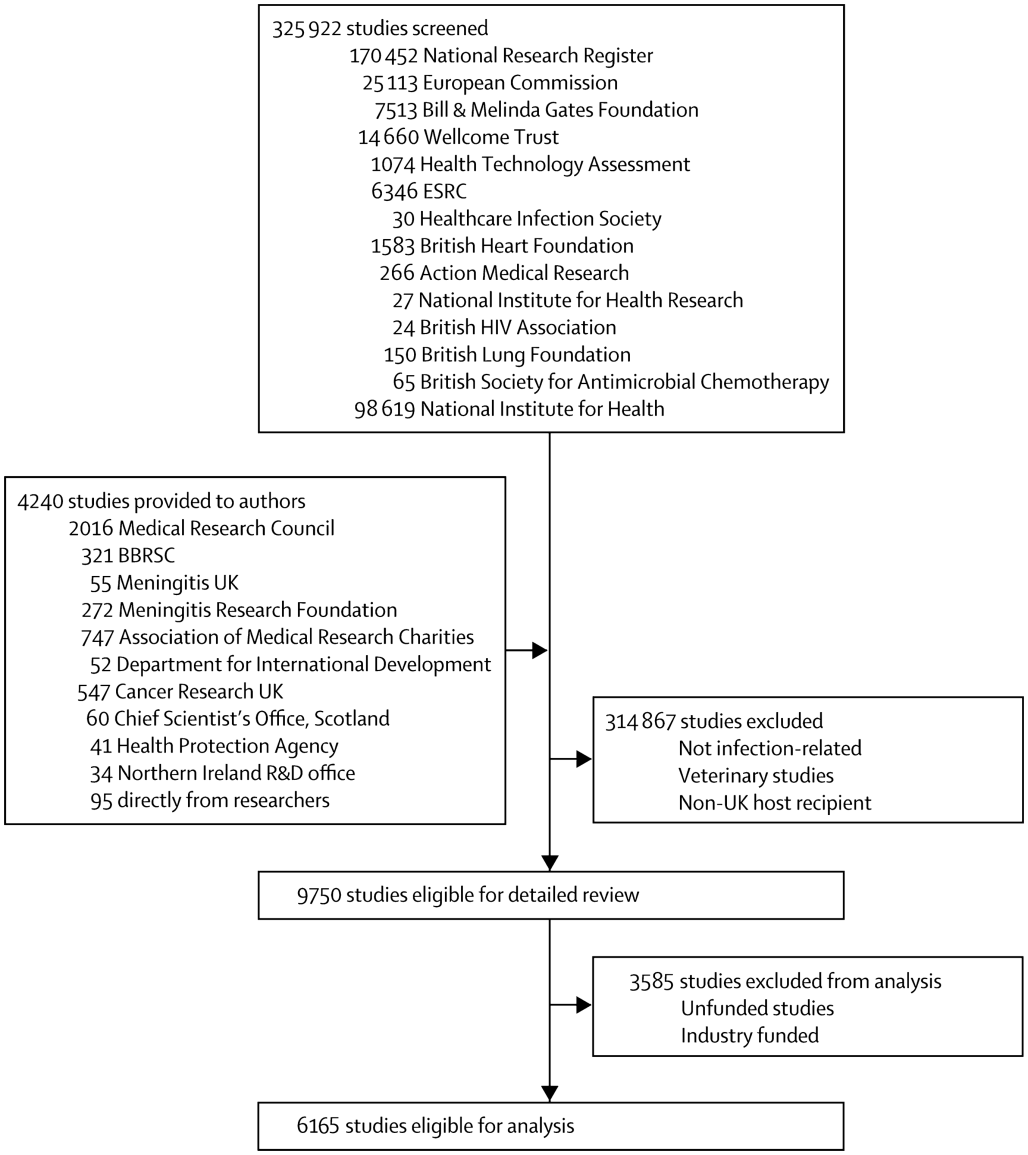
Sources and numbers of studies screened. BBSRC = Biotechnology and Biological Sciences Research Council. ESRC = Economic and Social Research Council. R&D = Research and Development. Total number of studies differs by n = 5 (0.08%) from previously published work following ongoing review of the data by the study team [2].

We obtained data from several sources for infectious disease research studies where funding was awarded between 1997 and 2010 (full list and further resources on methodology are openly available from http://researchinvestments.org/data). Figure 1 shows the sources of data and the numbers of studies explored at each stage of screening to reach the final set of studies for detailed analysis. We identified 6165 relevant studies for analysis. We assigned each study to primary disease categories. We outline the methodology for the categorisation of disease areas and classification of the funding sources, elaborated in detail previously.[2]

The overarching dataset was constructed by collating open-access data and directly contacting the major sources of public and philanthropic funding for infectious disease research studies, including the Wellcome Trust, Medical Research Council and other research councils, UK government departments, the European Commission, Bill and Melinda Gates Foundation, and other research charities. We also searched other databases, including Clinicaltrials.gov and the National Research Register. Within each category, we documented topic-specific subsections, including specific pathogen or disease. We allocated studies to one of four categories along the R&D continuum: pre-clinical; phases I, II or III; product development; and operational research (which includes epidemiological and implementation research). We developed nine major categories for funding organisations, based on total levels of research investment, and cross-referenced grants from funding organisation to disease categories and stage of R&D funding.

Global health studies include investments to UK institutions with a global partner organisation, or studies predominantly carried out or focused on a country other than the UK. Antimicrobial resistance includes antibacterial, antiviral, antifungal and antiparasitic studies. Reference to sexually transmitted infections excludes HIV/AIDS. Neglected tropical diseases (NTDs) were categorised based on the infections focused on by WHO (for the list of NTDs focused on by WHO see http://www.who.int/neglected_diseases/diseases/en). No private sector funding was included in this analysis as open-access data were limited.

Grants awarded in a currency other than pounds sterling were converted to UK pounds using the mean exchange rate in the year of the award (http://www.oanda.com/currency/average). All grant funding amounts were adjusted for inflation and reported in 2010 UK pounds.

We excluded studies not immediately relevant to infection, veterinary infectious disease research studies (unless there was a zoonotic component) those exploring the use of viral vectors to investigate non-communicable diseases, grants for symposia or meetings, or studies with UK contributions (e.g. as a collaborator), but the funding was awarded to a non-UK institution. Unfunded studies were excluded.

We used Microsoft Excel (versions 2000 and 2007) to categorize studies. Where needed, data were exported into Microsoft Access (versions 2000 and 2007) and specific keyword queries used to select precise sections of the data for analysis. We used Stata (version 11.0; StataCorp LP, Texas) for statistical analysis and to generate figures.

We then systematically analysed the investments by major funding organisations for research projects where a UK institution acted as a leading partner. For multi-centre collaborative studies, we included data where apportioned funding was indicated where UK institutions were the leading partner. For multi-centre collaborative studies where a UK institution was not a leading partner, we were unable to include the funding, which may represent an underestimate (particularly for studies led in the European Union or the United States). We used fold differences to measure the quantity of change in total investment, number of studies, mean grant, and median grant according to disease system, specific infection and funding organisation. We present median grants in the results section to minimize the effect of the skew from few, very large international project awards led by a UK institution.

We used nonparametric Mann-Whitney rank-sum test to assess the distribution of funding by funding source. Nonparametric K-sample test on equality of medians was applied to compare the median funding by funding source, and reported as a chi-squared statistic without Yates’ correction for continuity. Nonparametric Wilcoxon signed-rank test was applied when comparing matched data, such as time trends by funding source. The significance for all tests was defined at the 5% level (two-sided *P* = 0.05).

## Results

We identified 6165 funded studies in infectious disease research with total research investment of UK £2.6 billion. Of these, 2385 studies (38.7%) were investments by public research funding organisations totalling £1.4 billion (54%), 2874 studies (46.6%) by philanthropic funding organisations totalling £1.1 billion (42.4%).

Global health studies represented an investment of £928 million (35.7%). Overall, the mean amount of grant funding awarded was £421 733 (SD £1 315 935) and the median amount of grant funding awarded was £158 055 (IQR £49 490 – £352 699).

**Figure 2:**
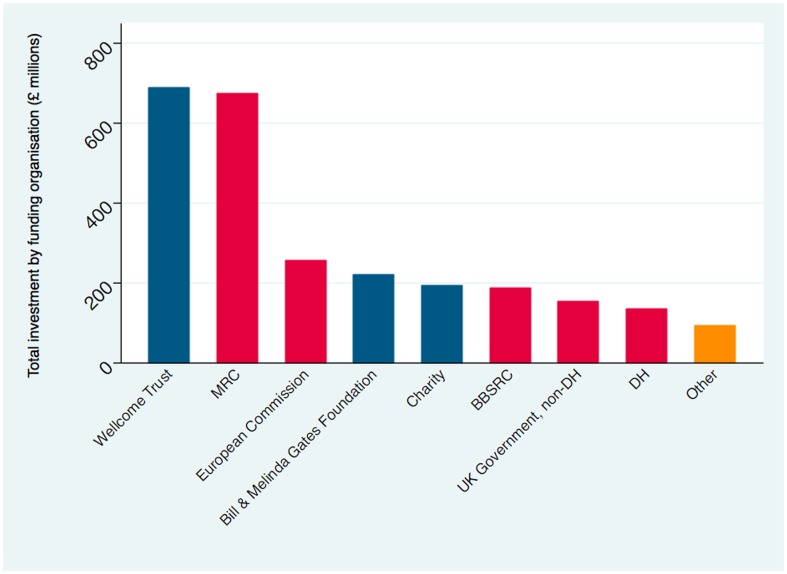
Investment in immunology and vaccine research by funding organisation. BBSRC = Biotechnology and Biological Sciences Research Council. DH = Department of Health. MRC = Medical Research Council. Blue designates philanthropic funding organisations. Red designates public funding organisations. Yellow designates other funding organisations.

Figure 2 shows the overall ranking of funding organisations by total research investment. The Wellcome Trust was the leading investor in infectious disease research with £688 million (26.5%), closely followed by the UK Medical Research Council (MRC) with £673 million (25.9%). Major funding organisations included the European Commission with £255 million (9.8%), the Bill and Melinda Gates Foundation with £220 million (8.5%) and the Biotechnology and Biological Sciences Research Council (BBSRC) with £186 million (7.2%). Charities and smaller foundations collectively accounted for £193 million of investment (7.4%) across 851 studies (13.8%).

**Figure 3:**
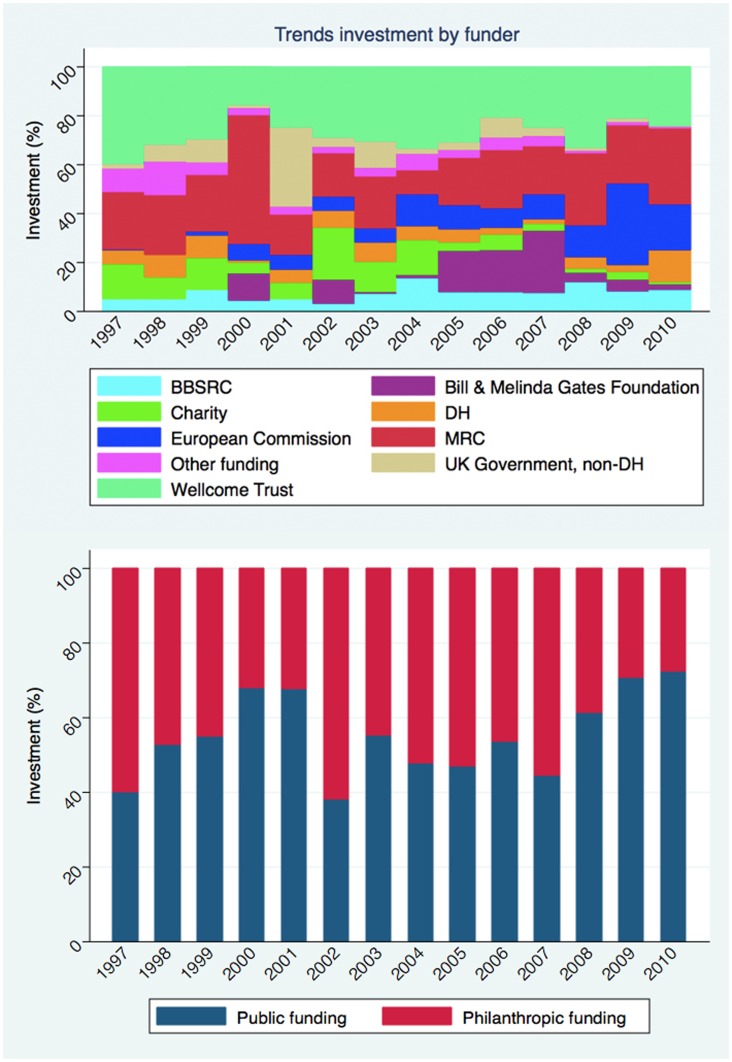
Trends in investment over time: a) stratified by funding organisation, b) stratified by public versus philanthropic funder. BBSRC = Biotechnology and Biological Sciences Research Council. DH = Department of Health. MRC = Medical Research Council.

Figure 3 shows the trends in research funding according to funding organisation over time. The Bill and Melinda Gates Foundation awarded the largest mean and median amount of grant funding at £5 664 699 (SD £8 966 093) and £1 488 432 (IQR £628 545 – £5 576 863), respectively.

**Figure 4:**
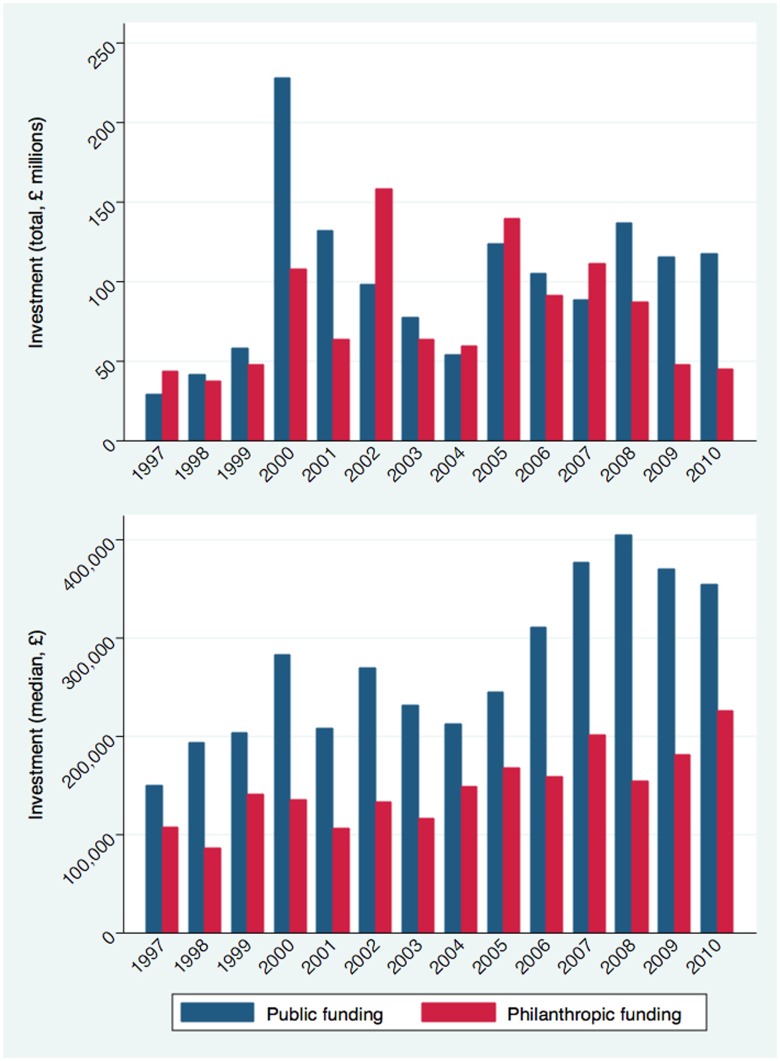
Trends in investment by public versus philanthropic funder over time: a) total research investment, b) median research investment.

**Figure 5:**
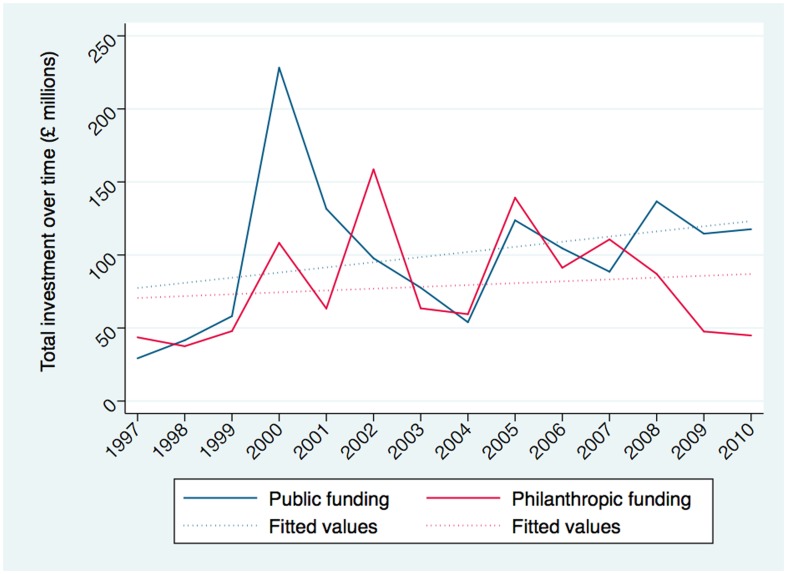
Fitted trend in investment by public versus philanthropic funder over time. Full line represents annual expenditure. Dotted line represents linear regression best-fit line over the 14-year study period.

Figure 4 compares the total investment and median amount of grant funding awarded by public and philanthropic investors. The public funding has been larger in 11 of the 14 years studied than philanthropic organisations, which accounts for a significant proportion of the funding ranging from 38% to 72% of total annual funding. Funding is volatile ranging, from ∼£40 million to ∼£160 million for the philanthropic organisations and ∼£30 million to ∼£230 million for the public funders. There is no obvious trend suggesting that the annual funding is increasing and has been fairly flat since 2005. The average median funding awarded by public organisations is larger at £255 992 (IQR £127 167 – £529 610), compared with £146 060 (IQR £52 433 – £286 518) for philanthropic organisation, almost two fold difference. Figure 5 shows an overall increase in research funding, greatest for public funding organisations. However the levels of funding are volatile over time, and the linear regression best-fit line should be interpreted with caution.

## 

### Funding along the research and development stages

Table 1 shows investment by funding source and research and development (R&D) stages. The funding for preclinical research accounted for the majority of investment with £1.6 billion (62.4%) with a median grant of £193 149 (IQR £74 157 – £365 587). Public investors funded 57.5% of the research with philanthropic investors funding 40.5%.

**Table 1:** Investment in immunology and vaccine research by funding source and research and development phase.

Funder	Investment (total); £ (%)	Studies (total); n (%)	Mean grant (total); £ (SD)	Fold difference	Median grant (total); £ (IQR)	Fold difference	Pre-clinical investment; £ (%)	Pre-clinical median grant; £ (IQR)	Fold difference	Pre-clinical investment; n (%)	Phase 1–3 investment; £ (%)	Phase 1–3 median grant; £ (IQR)	Fold difference	Phase 1–3 investment; n (%)	Product development investment; £ (%)	Product development median grant; £ (IQR)	Fold difference	Product development investment; n (%)	Operational research investment; £ (%)	Operational research median grant; £ (IQR)	Fold difference	Operational research investment; n (%)
Public funding	1,403,579,619	2,385	588,503	1.40	255,992	1.62	932,404,084	277,131	1.43	1,609	109,137,486	535,878	2.51	58	69,119,547	236,824	1.60	131	292,918,502	177,645	2.01	587
	54.0%	38.7%	1,447,668		127,167–529,610		66.4%	156,125–543,951		67.5%	7.8%	130,994–1,413,252		2.4%	4.9%	80,507–688,115		5.5%	20.9%	51,765–417,210		24.6%
BBSRC	186,268,429	578	322,264	0.76	253,398	1.60	186,243,256	253,479	1.31	576	0	0	0.00	0	0	0	0.00	0	25,173	12,586	0.14	2
	7.2%	9.4%	361,565		169,787–365,159		100.0%	176,508–365,951		99.7%	0.0%			0.0%	0.0%			0.0%	0.0%	812–24,359		0.3%
DH	134,961,745	285	473,550	1.12	203,544	1.29	14,317,188	215,773	1.12	28	6,840,563	808,336	3.79	7	20,373,031	291,554	1.98	40	93,430,963	175,134	1.98	210
	5.2%	4.6%	846,024		72,628–514,066		10.6%	57,538–556,546		9.8%	5.1%	140,714–1,712,753		2.5%	15.1%	101,275–707,126		14.0%	69.2%	72,294–394,057		73.7%
European Commission	255,015,533	219	1,164,454	2.76	439,762	2.78	187,782,118	195,999	1.01	164	0	0	0.00	0	12,680,401	624,297	4.23	14	54,553,014	555,497	6.30	41
	9.8%	3.6%	2,084,358		127,419–1,454,941		73.6%	119,659– 1,449,573		74.9%	0.0%			0.0%	5.0%	82298–1,504,880		6.4%	21.4%	382,254–1,454,941		18.7%
MRC	672,895,698	962	699,476	1.66	366,479	2.32	527,370,055	377,564	1.95	738	42,323,395	516,957	2.42	43	27,578,378	403,037	2.73	35	75,623,870	272,221	3.09	146
	25.9%	15.6%	993,012		199,287–713,178		78.4%	210,390–713,178		76.7%	6.3%	126,839–1,073,076		4.5%	4.1%	100,528–1,072,094		3.6%	11.2%	165719–562,944		15.2%
UK government, non-DH	154,438,214	341	452,898	1.07	110,178	0.70	16,691,467	121,493	0.63	103	59,973,528	1,833,607	8.59	8	8,487,737	127,733	0.87	42	69,285,483	81,566	0.92	188
	5.9%	5.5%	2,811,384		19,073–206,784		10.8%	19,851–206,784		30.2%	38.8%	84,889–3,059,723		2.3%	5.5%	39,707–242,762		12.3%	44.9%	15,547–194,524		55.1%
Philanthropic funding	1,102,469,932	2,874	383,601	0.91	146,060	0.92	656,630,852	154,734	0.80	2,056	27,387,963	114,168	0.53	62	50,456,146	168,035	1.14	109	367,994,932	80,028	0.91	647
	42.4%	46.6%	1,377,079		52,433–286,518		59.6%	67,894–283,391		71.5%	2.5%	22,900–355,370		2.2%	4.6%	64,854–438,110		3.8%	33.4%	18,846–253,342		22.5%
Bill & Melinda Gates Foundation	220,923,242	39	5,664,699	13.43	1,488,432	9.42	40,318,109	4,477,357	23.18	8	4,747,473	628,545	2.94	5	5,407,891	1,262,055	8.55	4	170,449,769	1,569,951	17.79	22
	8.5%	0.6%	8,966,093		628,545–5,576,863		18.2%	1,053,145–6,794,265		20.5%	2.1%	355,370–1,226,250		12.8%	2.4%	927,326–1,776,619		10.3%	77.2%			56.4%
Charity	193,459,157	851	227,332	0.54	87,318	0.55	130,381,297	103,052	0.53	525	4,993,262	56,010	0.26	40	3,620,490	65,676	0.44	35	54,464,109	51,531	0.58	251
	7.4%	13.8%	730,057		27,616–167,829		67.4%	48,134–190,307		61.7%	2.6%	14,252–131,102		4.7%	1.9%	9,423–168,035		4.1%	28.2%	11,000–116,503		29.5%
Wellcome Trust	688,087,494	1,984	346,818	0.82	168,434	1.07	485,931,447	178,375	0.92	1,523	17,647,229	348,687	1.63	17	41,427,765	289,781	1.96	70	143,081,054	107,626	1.22	374
	26.5%	32.2%	646,625		66,419–335,557		70.6%	81,881–315,033		76.8%	2.6%	116,470–1,660,204		0.9%	6.0%	95,888–551,291		3.5%	20.8%	28,364–340,503		18.9%
Other funding	93,936,339	906	103,683	0.25	28,626	0.18	33,510,841	21,004	0.11	372	10,301,944	213,471	1.00	25	13,303,136	38,625	0.26	95	36,820,418	30,531	0.35	414
	3.6%	14.7%	273,102		6,282–105,082		35.7%	5,917–81,491		41.1%	11.0%	23,805–305,339		2.8%	14.2%	10,000–1769,06		10.5%	39.2%	6,280–95,139		45.7%
Overall	2,599,985,851	6,165	421,733		158,055		1,622,545,777	193,149		4,037	146,827,393	213,471		145	132,878,829	147,621		335	697,733,852	88,232		1,648
			1,315,935		49,490–352,699		62.4%	74,157–365,587		65.5%	5.6%	53,116–839,713		2.4%	5.1%	38,625–409,663		5.4%	26.8%	18,513–250,423		26.7%
Table 1.																						

BBSRC = Biotechnology and Biological Sciences Research Council. DH = Department of Health. MRC = Medical Research Council.

Phase I, II, III clinical trials accounted for £147 million (5.6%) with the highest median grants at £213 471 (IQR £53 116 – £839 713). Public investors funded 73.4% of the research with philanthropic investors funding 18.7%. Industry funding, a major source of investment in clinical trials, could not be accurately sourced and was excluded from this analysis. Operational research accounted for £697 million (26.8%) with the lowest median grants at £88 232 (£18 513 – £250 423). Philanthropic investors funded 52.7% of the research with public investors funding 42.0%. Trends in investment over time by R&D pipeline is highlighted in figures S1 and S2.

Product development research accounted for the least investment with £133 million (5.1%) with a median grant of £147 621 (IQR £38 625 – £409 663). Public investors funded 52.0% of the research with philanthropic investors funding 38.0%.

Table 2 shows the ranking of funding organisation according to research type. The type of science funded by different organisations clearly varies according to the priorities of each funding organisation. The Wellcome Trust, MRC, European Commission, and BBSRC concentrated their investment on preclinical research (70.6%, 78.4%, 73.6% and 100%, respectively). The Bill and Melinda Gates Foundation, Department of Health and other UK government sources concentrated their research investment on operational research (77.2%, 69.2% and 44.9%, respectively).

**Table 2:** Ranking of investment in immunology and vaccine research by a) disease system, b) cross-cutting theme, and c) specific infectious disease.

Disease system	Top funder 1	Top funder 2	Top funder 3	Top funder 4	Top funder 5
Gastrointestinal infections	BBSRC	Wellcome Trust	MRC	European Commission	UK Government, non-DH
	33.1%	31.3%	13.4%	8.8%	4.7%
Haematological infections	Wellcome Trust	Bill & Melinda Gates Foundation	MRC	European Commission	Other funding
	34.2%	29.9%	18.4%	6.7%	10.9%
Hepatic infections	MRC	Wellcome Trust	DH	European Commission	BBSRC
	33.4%	19.0%	12.5%	11.7%	4.6%
Neglected tropical diseases	Wellcome Trust	Bill & Melinda Gates Foundation	MRC	European Commission	BBSRC
	45.2%	29.3%	14.8%	9.1%	3.8%
Neurological infections	DH	Wellcome Trust	MRC	Meningitis Research Foundation	Bill & Melinda Gates Foundation
	25.3%	24.9%	14.9%	12.1%	3.6%
Ocular infections	Wellcome Trust	MRC	Charity	UK Government, non-DH	DH
	63.3%	11.9%	7.4%	6.7%	6.4%
Respiratory infections	Wellcome Trust	MRC	European Commission	BBSRC	Department for International Development
	32.3%	29.6%	8.0%	6.2%	3.3%
Sexually-transmitted infections	MRC	DH	Cancer Research UK	Department for International Development	Wellcome Trust
	29.5%	19.8%	19.1%	9.1%	6.5%
HIV	MRC	Department for International Development	Wellcome Trust	European Commission	Bill & Melinda Gates Foundation
	33.8%	16.6%	15.8%	13.5%	7.5%
Overall	Wellcome Trust	MRC	European Commission	Bill & Melinda Gates Foundation	BBSRC
	26.47%	25.88%	9.81%	8.50%	7.16%

Disease system	Top funder 1	Top funder 2	Top funder 3	Top funder 4	Top funder 5
Diagnostics	DH	Cancer Research UK	European Commission	UK Government, non-DH	Wellcome Trust
	23.4%	17.0%	11.5%	11.2%	10.7%
Therapeutics	European Commission	Bill & Melinda Gates Foundation	Wellcome Trust	MRC	Department for International Development
	22.6%	18.5%	16.5%	16.0%	14.1%
Vaccines	MRC	Wellcome Trust	European Commission	Bill & Melinda Gates Foundation	BBSRC
	25.6%	21.4%	18.3%	12.2%	6.8%
Bacteriology	Wellcome Trust	MRC	BBSRC	European Commission	DH
	29.9%	21.8%	17.7%	8.2%	6.3%
Mycology	BBSRC	Wellcome Trust	MRC	European Commission	National Institute for Health
	30.5%	24.7%	16.6%	13.2%	6.2%
Parasitology	Wellcome Trust	Bill & Melinda Gates Foundation	MRC	European Commission	BBSRC
	41.2%	25.3%	15.2%	12.4%	2.8%
Prion	DH	European Commission	UK Government, non-DH	MRC	Other funding
	65.3%	11.3%	9.7%	6.2%	7.6%
Virology	MRC	Wellcome Trust	Cancer Research UK	European Commission	Department for International Development
	35.1%	18.1%	10.5%	8.7%	6.9%
Overall	Wellcome Trust	MRC	European Commission	Bill & Melinda Gates Foundation	BBSRC
	26.47%	25.88%	9.81%	8.50%	7.16%

Specific infection	Top funder 1	Top funder 2	Top funder 3	Top funder 4	Top funder 5
African trypanosomiasis	Wellcome Trust	MRC	Bill & Melinda Gates Foundation	European Commission	WHO
	75.4%	15.3%	8.8%	0.3%	0.1%
Aspergillus	National Institute for Health Research	Wellcome Trust	Fungal Research Trust	MRC	Other funding
	42.8%	35.4%	13.4%	7.4%	1.0%
Campylobacter	BBSRC	MRC	UK Government, non-DH	Wellcome Trust	Other funding
	82.2%	6.5%	6.1%	2.7%	2.5%
Candida	National Institute for Health Research	Fungal Research Trust	Dunhill Medical Trust		
	88.0%	9.2%	2.7%		
Chagas disease	Wellcome Trust	MRC	European Commission		
	57.3%	23.5%	19.2%		
Chlamydia	Department of Health	UK Government, non-DH	MRC	Wellcome Trust	BUPA Foundation
	49.9%	15.9%	13.5%	7.9%	4.0%
Clostridium	BBSRC	European Commission	Wellcome Trust	MRC	National Institute for Health Research
	24.3%	20.3%	19.5%	17.1%	12.6%
CMV	MRC	Wellcome Trust	NHS	Bill & Melinda Gates Foundation	National Institute for Health
	65.5%	24.7%	5.6%	1.3%	0.8%
Dengue	Bill & Melinda Gates Foundation	Wellcome Trust	MRC	European Commission	BBSRC
	76.6%	12.5%	7.6%	2.6%	0.8%
Diphtheria	European Commission	UK Government, non-DH			
	84.5%	15.5%			
E. coli	BBSRC	Wellcome Trust	MRC	Economic and Social Research Council	European Commission
	40.9%	39.0%	5.8%	5.1%	3.8%
EBV	Cancer Research UK	MRC	Wellcome Trust	BBSRC	Other funding
	55.9%	25.7%	14.7%	0.9%	2.7%
Gonorrhoea	Wellcome Trust	BBSRC	UK Government, non-DH	NHS	Other funding
	48.9%	25.6%	15.5%	4.4%	5.6%
Helicobacter	MRC	Cancer Research UK	Wellcome Trust	NHS	Other funding
	33.6%	28.8%	12.2%	5.7%	19.8%
Helminths	Wellcome Trust	Bill & Melinda Gates Foundation	MRC	BBSRC	European Commission
	53.7%	20.0%	14.0%	7.3%	4.5%
Hepatitis B	MRC	Wellcome Trust	European Commission	National Blood Service	Department of Health
	43.0%	23.3%	7.2%	4.4%	3.6%
Hepatitis C	MRC	Wellcome Trust	DH	European Commission	UK Government, non-DH
	35.1%	17.1%	14.3%	12.8%	4.5%
HIV	MRC	Department for International Development	Wellcome Trust	European Commission	Bill & Melinda Gates Foundation
	33.8%	16.6%	15.8%	13.5%	7.5%
HPV	Cancer Research UK	MRC	DH	European Commission	Wellcome Trust
	50.8%	24.4%	9.2%	4.9%	3.4%
HSV	MRC	Wellcome Trust	UK Government, non-DH	BBSRC	Other funding
	65.8%	23.7%	5.4%	3.2%	2.1%
Influenza	MRC	BBSRC	European Commission	Wellcome Trust	Other funding
	52.9%	16.6%	12.9%	12.7%	4.9%
Leishmaniasis	Wellcome Trust	European Commission	MRC	BBSRC	
	42.5%	35.3%	18.2%	4.0%	
Leprosy	Wellcome Trust				
	100.0%				
Listeriosis	BBSRC	Wellcome Trust	European Commission	MRC	
	40.6%	26.2%	21.7%	11.6%	
Lymphatic filariasis	Bill & Melinda Gates Foundation	MRC	Wellcome Trust		
	95.5%	3.9%	0.6%		
Malaria	Wellcome Trust	Bill & Melinda Gates Foundation	MRC	European Commission	UK Government, non-DH
	34.5%	26.1%	19.1%	13.0%	3.9%
Measles	MRC	European Commission	UK Government, non-DH	DH	Wellcome Trust
	49.2%	35.5%	7.3%	5.3%	2.7%
Meningitis	Wellcome Trust	Meningitis Research Foundation	MRC	DH	Meningitis UK
	27.4%	22.3%	21.2%	7.3%	4.4%
Norovirus	Wellcome Trust	European Commission	MRC	DH	
	47.7%	40.6%	9.9%	1.8%	
Onchocerciasis	European Commission	Wellcome Trust			
	75.5%	24.5%			
Pertussis	Wellcome Trust	UK Government, non-DH	MRC	Other funding	
	46.8%	31.0%	19.5%	2.8%	
Polio	MRC	UK Government, non-DH			
	98.5%	1.5%			
Pseudomonas	MRC	BBSRC	Wellcome Trust	DH	UK Government, non-DH
	33.2%	27.6%	10.8%	7.4%	6.9%
Rotavirus	Wellcome Trust	European Commission	BBSRC	MRC	Other funding
	57.9%	20.7%	11.2%	6.1%	4.1%
RSV	Wellcome Trust	MRC	BBSRC	UK Government, non-DH	Other funding
	44.8%	38.5%	5.3%	5.2%	6.3%
Salmonella	BBSRC	Wellcome Trust	MRC	European Commission	Other funding
	55.0%	18.4%	17.8%	8.1%	0.7%
Schistosomiasis	Bill & Melinda Gates Foundation	Wellcome Trust	European Commission	BBSRC	Other funding
	66.2%	22.9%	8.8%	1.3%	0.8%
Shigella	Wellcome Trust	European Commission	MRC	National Institute for Health	
	41.7%	25.3%	20.0%	13.1%	
Syphilis	Wellcome Trust	MRC	DH	Terence Higgins Trust	
	53.5%	21.7%	19.5%	5.3%	
Tetanus	European Commission	Wellcome Trust			
	83.6%	16.5%			
Trachoma	MRC				
	100.0%				
Tuberculosis	Wellcome Trust	MRC	Department for International Development	European Commission	Bill & Melinda Gates Foundation
	40.3%	23.2%	8.9%	7.8%	6.3%
VZV	MRC	DH	Wellcome Trust	Other funding	
	45.9%	22.5%	15.5%	16.1%	
Overall	Wellcome Trust	MRC	European Commission	Bill & Melinda Gates Foundation	BBSRC
	26.47%	25.88%	9.81%	8.50%	7.16%

BBSRC = Biotechnology and Biological Sciences Research Council. DH = Department of Health. MRC = Medical Research Council. CMV = Cytomegalovirus. EBV = Epstein-Barr virus. HIV = Human immunodeficiency virus. HPV = Human Papillomavirus. HSV = Herpes Simplex virus. RSV = Respiratory Syncytial virus. VZV = Varicella Zoster virus.

### Infectious disease system

Table S1 shows investment by infectious disease system, and specific infection.

The total funding for HIV-related research projects was the greatest with £478 million (18.4%) followed by respiratory infections with £419 million (16.1%), haematological infections with £413 million (15.9%). Gastrointestinal infections received £249 million (9.6%) and NTDs received £230 million (8.8%). Public investors accounted for the majority of research funding for HIV, gastrointestinal, hepatic, respiratory, and sexually transmitted infections. In contrast, philanthropic investors accounted for the majority of research funding for NTDs, haematological infections (primarily malaria) and ophthalmic infections. Largest mean grants were awarded to HIV at £625 073 (SD £2 276 762), with grants by public investors 2.46 fold greater than philanthropic investors. Largest median grants were awarded to NTDs at £248 750 (IQR £91 196–£451 453), with grants by public investors 1.75 fold greater than philanthropic investors.

### Specific infection

Several infections are highly supported by public funding sources compared with philanthropic support. Notable examples include influenza with a 6.38 fold difference (£67.8 million versus £10.6 million), chlamydia with a 9.02 fold difference (£17.7 million versus £2.0 million), campylobacter with a 32.87 fold difference (£22.8 million versus £0.7 million), and salmonella with a 4.39 fold difference (£45.0 million versus £10.2 million). Conversely, philanthropic funding sources greatly outweigh public funding for African Trypanosomiasis (£36.0 million versus £6.7 million), lymphatic filariasis (£45.2 million versus £1.8 million), schistosomiasis (£36.3 million versus £4.4 million), meningitis (£35.3 million versus £16.3 million) and EBV (£32.3 million versus £12.1 million).

### Cross-cutting theme

Novel technologies to fight infection played an important role in infectious disease research funding. Diagnostics research accounted for £100 million of investment (3.9%), primarily by the Department of Health (23.4%), Cancer Research UK (17.0%) and the European Commission (11.5%). Therapeutics research accounted for £408 million (15.7%), primarily by the European Commission (22.6%), Bill and Melinda Gates Foundation (18.4%) and the Wellcome Trust (16.5%). Vaccine research accounted for £235 million (9.0%), with major funders being the MRC (25.6%), Wellcome Trust (21.4%) and the European Commission (18.3%). Trends in investment over time by technologies to tackle infectious diseases are highlighted in figures S1 and S2. According to type of microbiological organism, viruses were the major area of funding with £1.0 billion (39.5%) followed by parasites with £667 million (25.7%), and bacteria with £588 million (22.6%). Fungal research attracted £48 million (1.9%) and prion research attracted £34 million (1.3%), primarily from the Department of Health. All microbiological categories were funded primarily by public investors, with exception of parasitology where 67.1% of funding came from philanthropic investors.

## Discussion

We present the first study to systematically analyse the investment by funding organisations for infectious disease research. Funding trends over time highlight the disparities in funding amounts and stage of funding between funding organisations and the infectious diseases they fund. Studies with a clear global health focus, i.e. those performed outside of the UK, in partnership with an international collaborator, or studying a disease primarily affecting a low-income setting, represented 35.7% of total investment (£928 million).

Funding trends over time show that charitable funding declines dramatically from 2007, suggesting that the financial crisis adversely impact on health research funding, in particular from smaller charities that were affected by the economic crisis. The volatility of charitable funding is evident, both as a proportion of total investment as well as the sum of total investment. In contrast, funding for infectious disease research was stable, as a proportion of funding and as total investment, for the large funding organisations such as the Wellcome Trust and MRC. The funding landscape also appears to be shifting. These data highlight the dependence on these two leading funders for health research. In addition, the Bill and Melinda Gates Foundation contribute substantially to infectious disease research funding, interjecting a small number of grants of great monetary value. The European Commission emerges as a major funder, particularly from the year 2000.

Contrasting public and philanthropic funding, total investment is consistently greater from public sources, both in terms of total investment and median investment. Analysing these data over time, the funding from public and philanthropic sources does not appear to be equalising. Fitted values (figure 5) for public and philanthropic funding do show a trend towards increased financing for research overall. However, a drop in philanthropic funding, attributed to the smaller charitable organisations, is clearly apparent from 2007. It will be important to follow up research investments over the coming years to see whether these trends have reverted.

Of note, there is a lack of industry funding from the data. This is primarily due to the methodological decision to exclude industry funding from the analysis, as the open access data available on R&D investments of pharmaceutical firms clearly underestimated the contribution of industry to infectious disease research. Industry partners are likely to contribute more towards clinical trials such as phase 1, phase 2, and phase 3 studies. In order to make evidence-informed decisions, we require complete and accurate data. The pharmaceutical industry should work alongside public and philanthropic funders in order to map, monitor, and evaluate research funding. Publishing such data online in an open access database, such as on www.researchinvestments.org, will be mutually beneficial to both academic institutions and pharmaceutical and biotech companies. In addition, it is unlikely that divulging past and current research investments would jeopardise research in progress. On the contrary, understanding the emerging horizons in current research would allow complementary studies to be performed, and the development of the research base.

We currently lack informative data on the distribution of studies along the research and development pipeline.[10], [12] Data from this analysis shows the weight of preclinical research on funding. The great majority of funding is allocated to preclinical work, with a new minority allocated to phase 1, 2, 3 trials and product development. Operational research, which includes epidemiological studies, attracted the second greatest level of funding. This is particularly strong in global health studies.[13]–[14] This trend does not appear to change drastically over time.

We require innovative health financing, and new funding streams to promote innovative delivery of new tools to fight infectious diseases and address the burden of disease.[15]–[18] Funding allocated for tools to tackle infectious diseases on the other hand is more volatile. There is a clear surge in investment from 2000. This investment wanes slightly over the years until further boosts are made from the year 2005. Most of the investment is allocated to therapeutics or studies involving drugs. Vaccine research receives considerable investment, however this tends to be concentrated according to intermittent funding streams.[19] Diagnostics research appears to be the least well funded of the tools for infectious disease control.

This study maps the research funding landscape for infectious disease research in the UK. The UK is the second greatest funder for global health, after the US.[20]–[21] It is essential that we understand the funding contributions from other countries, both from the major world economies of the G20 as well as the investments made by local governments and NGOs in low-income settings.[22] An example of a national system that promotes transparency is the extensive online database on the “Research Portfolio Online Reporting Tools” (RePORT) website by the NIH Research, Condition, and Disease Categorization system (http://report.nih.gov/catego rical_spending.aspx). In addition to a lack of openness with industry funding, there is a lack of openness for large grants awarded to international consortia. In the case of these consortia, although the total grant is often well documented, the international transactions between the lead institution and partner institutions are less well documented.

This work has major implications for academic institutions, governments, funding organisations, and policy makers. Particularly with regards to public funding, there is a duty to invest scarce resources wisely.[23] However, if governments, policy-makers and executives of the funding organisations are allocating resources without accurate knowledge of the current funding terrain, there is bound to be inefficiency.

We urge funding organisations to share data online so that trends in funding may be appropriately assessed. RESIN: Research Investments in Global Health (www.researchinvestments.org) is an initiative that aims to act as an open access portal to facilitate documentation of investments in health research. Inequities in research funding have major implications for global health. Simple measures such as documentation and dissemination of data may act to redress these inequities.

## Supporting Information

Figure S1 Trends in investment over time: a) stratified by research and development phase, b) stratified by infectious disease tool.
(JPG)

Figure S2 Proportion of investment over time: a) stratified by research and development phase, b) stratified by infectious disease tool.
(JPG)

Table S1 Investment in immunology and vaccine research by a) specific infectious disease and b) disease system. CMV = Cytomegalovirus. EBV = Epstein-Barr virus. HIV = Human immunodeficiency virus. HPV = Human Papillomavirus. HSV = Herpes Simplex virus. RSV = Respiratory Syncytial virus. VZV = Varicella Zoster virus.
(XLS)

## References

[1] Atun R, Knaul FM, Akachi Y, Frenk J (2012) Innovative financing for health: what is truly innovative? Lancet 380: 2044–2049. 23102585 10.1016/S0140-6736(12)61460-3

[2] Head MG, Fitchett JR, Cooke MK, Wurie FB, Hayward AC, et al (2013) UK investments in global infectious disease research 1997–2010: a case study. Lancet Infect Dis 13: 55–64. 23140942 10.1016/S1473-3099(12)70261-X

[3] Fisk NM, Atun R (2009) Systematic analysis of research underfunding in maternal and perinatal health. BJOG 116: 347–356. 19187366 10.1111/j.1471-0528.2008.02027.x

[4] Moran M, Guzman J, Henderson K, Liyanage R, Wu L, et al. (2012) G-FINDER – Neglected disease research and development: A five year review. Sydney: Policy Cures. Available: http://www.policycures.org/downloads/GF2012_Report.pdf. Accessed 12 October 2013

[5] Gross CP, Anderson GF, Powe NR (1999) The relation between funding by the National Institutes of Health and the burden of disease. N Engl J Med 340: 1881–1887. 10369852 10.1056/NEJM199906173402406

[6] Gillum LA, Gouveia C, Dorsey ER, Pletcher M, Mathers CD, et al (2011) NIH disease funding levels and burden of disease. PLoS One 6: e16837. 21383981 10.1371/journal.pone.0016837 PMC3044706

[7] Kyratsis Y, Ahmad R (2013) Mapping the terrain of investment in global infectious diseases. Lancet Infect Dis 13: 6–7. 23140943 10.1016/S1473-3099(12)70289-X

[8] WHO (2012) Research and Development to Meet Health Needs in Developing Countries: Strengthening Global Financing and Coordination. Geneva: World Health Organization. Available: http://www.who.int/phi/CEWG_Report_5_April_2012.pdf. Accessed 12 October 2013

[9] Røttingen J-A, Chamas C (2012) A new deal for global health R&D? The recommendations of the Consultative Expert Working Group on Research and Development (CEWG). PLoS Med 9: e1001219. 22615545 10.1371/journal.pmed.1001219 PMC3352865

[10] Røttingen J-A, Regmi S, Eide M, Young AJ, Viergever RF, et al (2013) Mapping of available health research and development data: what’s there, what's missing, and what role is there for a global observatory? Lancet 382: 1286–1307. 23697824 10.1016/S0140-6736(13)61046-6

[11] WHO (2012) Draft working paper: A global health R&D observatory – developing a case for its development. Geneva: World Health Organization. Available: http://www.who.int/phi/documents/dwp1_global_health_rd_observatory_16May13.pdf. Accessed 12 October 2013

[12] Terry RF, Allen L, Gardner CA, Guzman J, Moran M, et al (2012) Mapping global health research investments, time for new thinking-a Babel Fish for research data. Health Res Policy Syst 10: 28. 22938160 10.1186/1478-4505-10-28 PMC3471018

[13] Zachariah R, Ford N, Maher D, Bissell K, Van den Bergh R, et al (2012) Is operational research delivering the goods? The journey to success in low-income countries. Lancet Infect Dis 12: 415–421. 22326018 10.1016/S1473-3099(11)70309-7

[14] Cobelens F, van Kampen S, Ochodo E, Atun R, Lienhardt C (2012) Research on implementation of interventions in tuberculosis control in low- and middle-income countries: a systematic review. PLoS Med 9: e1001358. 23271959 10.1371/journal.pmed.1001358 PMC3525528

[15] Stuckler D, King L, Robinson H, McKee M (2008) WHO’s budgetary allocations and burden of disease: a comparative analysis. Lancet 372: 1563–1569. 18984189 10.1016/S0140-6736(08)61656-6 PMC7159087

[16] Katz I, Komatsu R, Low-Beer D, Atun R (2011) Scaling up towards international targets for AIDS, tuberculosis, and malaria: contribution of global fund-supported programs in 2011–2015. PLoS One 6: e17166. 21383839 10.1371/journal.pone.0017166 PMC3044165

[17] Lal SS, Uplekar M, Katz I, Lonnroth K, Komatsu R, et al (2011) Global Fund financing of public-private mix approaches for delivery of tuberculosis care. Trop Med Int Health 16: 685–692. 21332892 10.1111/j.1365-3156.2011.02749.x

[18] Katz I, Aziz MA, Olszak-Olszewski M, Komatsu R, Low-Beer D, et al (2010) Factors influencing performance of Global Fund-supported tuberculosis grants. Int J Tuberc Lung Dis 14: 1097–1103. 20819253

[19] Wolfson LJ, Gasse F, Lee-Martin SP, Lydon P, Magan A, et al (2008) Estimating the costs of achieving the WHO-UNICEF Global Immunization Vision and Strategy, 2006–2015. Bull World Health Organ 86: 27–39. 18235887 10.2471/BLT.07.045096 PMC2647343

[20] Dorsey ER, de Roulet J, Thompson JP, Reminick JI, Thai A, et al (2010) Funding of US biomedical research, 2003–2008. JAMA 303: 137–143. 20068207 10.1001/jama.2009.1987 PMC3118092

[21] Dorsey ER, George BP, Dayoub EJ, Ravina BM (2011) Finances of the publishers of the most highly cited US medical journals. J Med Libr Assoc 99: 255–258. 21753918 10.3163/1536-5050.99.3.013 PMC3133891

[22] McCoy D, Chand S, Sridhar D (2009) Global health funding: how much, where it comes from and where it goes. Health Policy Plan 24: 407–17. 19570773 10.1093/heapol/czp026

[23] Head MG, Fitchett JR, Atun R (2013) Global health priorities and research funding - Authors’ reply. Lancet Infect Dis 13: 653. 10.1016/S1473-3099(13)70169-5 23886328

