# Supplementary figures and images for: Funding Infectious Disease Research: A Systematic Analysis of UK Research Investments by Funders 1997–2010
Source: PLoS One. 2014 Aug 27;9(8):e105722. doi: 10.1371/journal.pone.0105722 (PMC4146508; doi:10.1371/journal.pone.0105722)

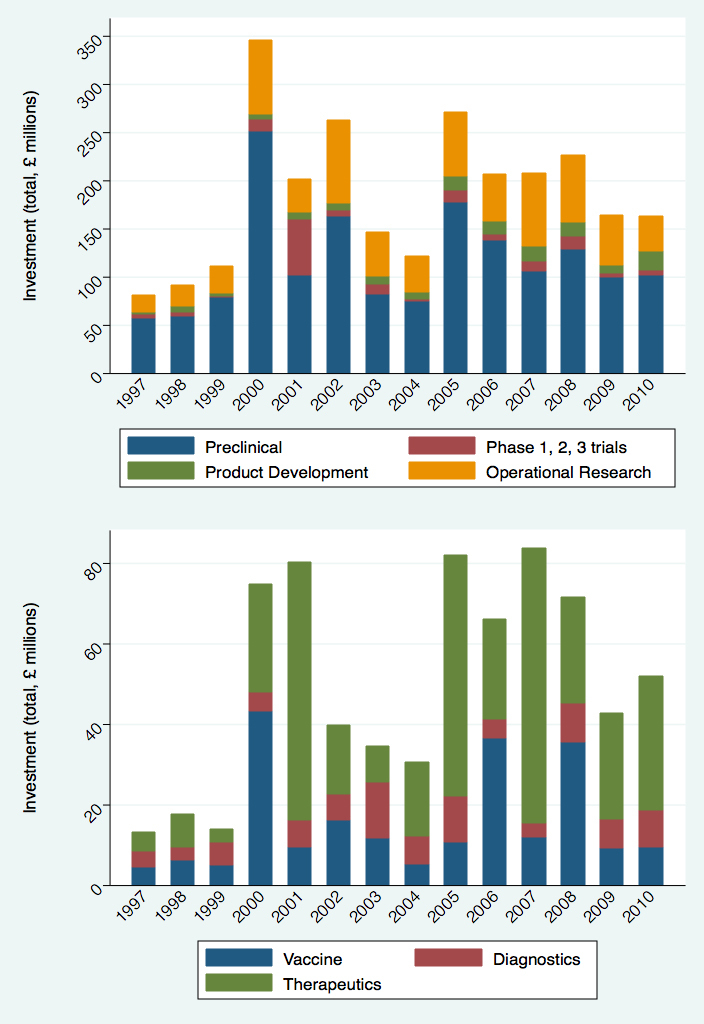

Supplement: Figure S1 — Trends in investment over time: a) stratified by research and development phase, b) stratified by infectious disease tool. (JPG) [file pone.0105722.s001.jpg]

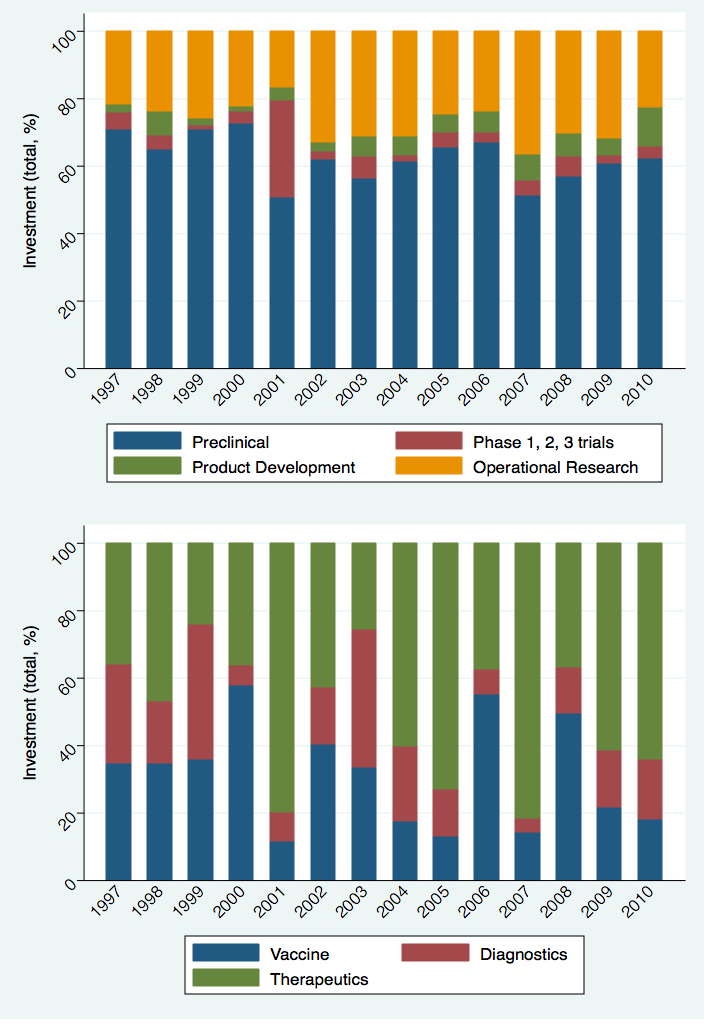

Supplement: Figure S2 — Proportion of investment over time: a) stratified by research and development phase, b) stratified by infectious disease tool. (JPG) [file pone.0105722.s002.jpg]
